# Whippets have larger abdominal caudal vena cava to aorta ratios than other dogs on computed tomography

**DOI:** 10.3389/fvets.2026.1971485

**Published:** 2026-10-23

**Authors:** Shanti Lucie Esmieu, Man-Hei Ma, Panagiotis Mantis, Silvia Biondo, Radu Constantinescu, Ana Rita Ramalho Furtado

**Affiliations:** 1 Dick White Referrals, Cambridgeshire, United Kingdom; 2 Veterinary Diagnostic Imaging Consultants Limited, Newmarket, Suffolk, United Kingdom

**Keywords:** caudal vena cava to aorta ratio, computed tomography, general anaesthesia, intravascular volume, mean ± 2 SD, whippet

## Abstract

**Introduction:**

Whippets are sighthounds with distinct cardiovascular adaptations associated with athletic performance. The caudal vena cava to aorta ratio (CVC/Ao) is an established index of intravascular volume status, assessed ultrasonographically and on computed tomography (CT) at thoracic and abdominal sites, but breed-specific differences remain unreported. We compared thoracic and abdominal CVC/Ao in whippets and non-whippet controls, and with published reference values, and hypothesised that whippets would have higher ratios.

**Methods:**

Sixty-eight dogs without known cardiovascular disease (34 whippets, 34 non-whippet controls) were retrospectively reviewed. CTs were performed under general anaesthesia, with CVC/Ao measured at standardised abdominal and thoracic sites. Groups were compared with Welch’s two-sample *t*-tests, sites within animals with paired t-tests, and analysis of covariance adjusted for weight and age. Significance was set at *p* < 0.05.

**Results:**

Whippets had significantly higher abdominal CVC/Ao than controls (1.54 ± 0.29 vs. 1.28 ± 0.23; *p* < 0.001), whereas thoracic ratios did not differ (1.22 ± 0.23 vs. 1.28 ± 0.26; *p* = 0.29). The abdominal difference persisted after adjustment for weight and age (adjusted difference 0.29, 95% CI 0.15–0.42; *p* < 0.001). Within whippets, abdominal ratios exceeded thoracic ratios (*p* < 0.001), a difference not seen in controls (*p* = 0.99). The thoracic ratio declined with age independently of breed and weight (−0.023 per year; *p* = 0.002). Both groups exceeded published CT values at both sites, and a greater proportion of whippets than controls exceeded the ultrasonographic upper limit (73.5% vs. 41.2%; *p* = 0.007). Observed mean ± 2 SD limits were 0.96–2.13 (abdomen, whippets), 0.82–1.75 (abdomen, controls) and 0.76–1.74 (thorax, combined); these are cohort observations rather than breed-specific reference ranges.

**Discussion:**

Whippets had larger abdominal CVC/Ao than controls, with comparable thoracic ratios, indicating a site-specific increase not attributable to body size or age. Because both groups exceeded published values, reference values are not transferable across imaging modality irrespective of breed or anaesthetic status. Modality-, breed- and possibly age-specific ranges are needed, and we provide preliminary limits as a starting point.

## 1 Introduction

The inferior vena cava to aorta ratio (IVC/Ao) was first described in human paediatrics on ultrasound (1, 2), to assess body fluid status in emergency settings, and was later extrapolated to canine patients as caudal vena cava to aorta ratio (CVC/Ao) (3, 4). The wide variability in body size made it difficult to establish normal absolute values for IVC diameter in humans and CVC dimensions in dogs, and therefore this ratio was introduced (1, 3, 5). The CVC/Ao has also been investigated on computed tomography (CT) in sedated dogs to establish a thoracic reference range (9), as well as in dogs under general anaesthesia with different thoracic, abdominal and systemic diseases (6). However, breed specific values for this ratio have not been reported yet. Whippets are sighthounds renowned for their sprint athletic performances, resulting in specific cardiovascular adaptations (7, 8), which may in turn affect vascular dimensions.

The aims of this study were to compare thoracic and abdominal CVC/Ao measured on CT between whippets and non-whippet control dogs without known cardiovascular disease; to compare these values with published CT and ultrasonographic reference ranges; and to derive breed-specific mean ± 2 SD limits for both groups. We hypothesised that whippets would have higher CVC/Ao than non-whippet breeds.

## 2 Materials and methods

### 2.1 Patient selection and description

This was a retrospective, descriptive, observational study. The clinical database and Picture Archiving and Communications System (PACS) of the presenting institution were initially searched for whippets who had undergone CT of any region between October 2011 and July 2025. Amongst these, patients were included only if they had a CT study of the thorax (extending at least up to the mid right kidney) and/or of the abdomen (which included the caudal thorax) for clinical reasons unrelated to suspected cardiovascular disease (including congenital or acquired vascular anomalies). Patients were also excluded if a major complication occurred during general anaesthesia. Dogs were not excluded on the basis of intra-abdominal or other non-cardiovascular disease.

The clinical database was searched again for patients who had undergone a CT study of the same region(s) and non-whippet patients without a known cardiovascular disease were randomly selected to constitute a control group for comparison. For each group, the following data was collected from their medical records: signalment (age, breed, sex and weight) and diagnosis at the time of the CT study.

### 2.2 CT imaging and general anaesthesia

Two different CT scanners were used in different proportions in each group: a 16-slice helical scanner (PNMS MX16 Slice, Philips Neusoft Medical Systems, Shenyang, China) prior to July 2023 and a 64-slice helical scanner (GE Revolution EVO (64 slices), GE Healthcare, Cincinnati, OH, United States) after July 2023. Most whippets (22/34,65%) were scanned on the 16-slice helical CT scanner and the remaining were scanned on the 64-slice scanner. The CT images for all but three control dogs (31/34, 91%) were obtained with the GE Revolution EVO CT scanner, while the others were scanned with the 16-slice scanner. All studies were acquired at 120 kVp. On the 16-slice scanner, tube current was 236–288 mA with a pitch of 0.86 in whippets and 0.75 in controls. On the 64-slice scanner, settings were 200 mA and a pitch of 0.51 in whippets, and 190–220 mA with a pitch of 0.51–0.53 in controls.

For all dogs, images were reconstructed in high and low frequency spatial algorithms with overlapping transverse slices between 1 and 2.5 mm thick (depending on the size of the patient and the region(s) scanned). Slice thickness was determined by patient body weight rather than by the scanner used, with the 2.5 mm thickness applied only to patients weighing more than 40 kg; larger patients are therefore expected to have correspondingly larger vessels, mitigating the partial volume effect.

When acquired, data was also obtained 60 s following an intravenous (IV) injection of iodinated non-ionic contrast medium (Iohexol^1^), which included the relevant portions of the thoracic and abdominal CVC and Ao. Most whippets (82%) also had post contrast DICOM images (Digital Imaging and Communications in Medicine) visualisation software: OsiriX (Pixmeo SARL, Bernex, Switzerland; version 14.2.2) available for the same pre-contrast region(s). All control dogs had an IV injection of the contrast medium and DICOM images available of the same pre-contrast region(s).

All CT studies were performed in sternal recumbency under general anaesthesia. Premedication and induction protocols were tailored to each patient by the anaesthetist (agents included dexmedetomidine^2^, methadone^3^, butorphanol^4^, alfaxalone^5^, propofol^6^, ketamine^7^ and diazepam^8^, among others), and anaesthesia was maintained with isoflurane^9^ in oxygen. A breath-hold was used to minimise diaphragmatic motion artefact. The breath-hold technique was protocolised by scanner era rather than by breed: dogs scanned on the 16-slice scanner underwent manual lung recruitment followed by an end-expiratory apnoeic pause maintained with a positive end-expiratory pressure (PEEP) valve, whereas dogs imaged on the 64-slice scanner were held under positive-pressure mechanical ventilation with an inspiratory positive-pressure hold. Both techniques were therefore performed under positive intrathoracic pressure. Because scanner use was unevenly distributed between the groups, this breath-hold technique was also largely group-linked, with the end-expiratory technique used in most whippets and the inspiratory-hold technique in most controls, although premedication and induction agents remained individualised. Actual airway pressures were not recorded in this retrospective cohort.

On admission, the owner of each dog signed a detailed consent form that specifically included the use of anonymised clinical data and images in research projects such as this one.

### 2.3 Measurements

All CT studies were retrieved to a computer with DICOM, and measurements were taken by an observer who was not blinded to breeds and group assignment. Short-axis diameters were obtained on transverse plane from low frequency spatial algorithms (soft tissue windows) at standardised sites (6, 9). The thoracic CVC and Ao measurements were taken at the same level, between the heart and the diaphragm (not in contact with either), where the vessels were most sharply defined (Figures 1, 2). Measurements in the abdomen were taken between the post-renal segment and porta hepatis for the CVC and just cranial to the exit of the coeliac artery for the Ao on the same slice, where the vessels were best defined and as isolated from surrounding structures as possible (Figures 3, 4). In patients where only one of the two regions was scanned, the portion of the other region included in the field of view was sufficient for taking relevant measurements (cranial abdomen for thoracic scan and caudal thorax for abdominal scan). Measurements were taken on post contrast images when available, and on pre-contrast images otherwise, avoiding points of motion or volume averaging artefacts. Measurement reliability was assessed in a random subset of 20 dogs (10 whippets, 10 controls) for both anatomical sites. A second observer, blinded to breed and group assignment, measured thoracic and abdominal CVC and Ao diameters for each dog on two separate occasions, with images cropped to small regions of interest displaying only these vessels obscuring body conformation.

**Figure 1:**
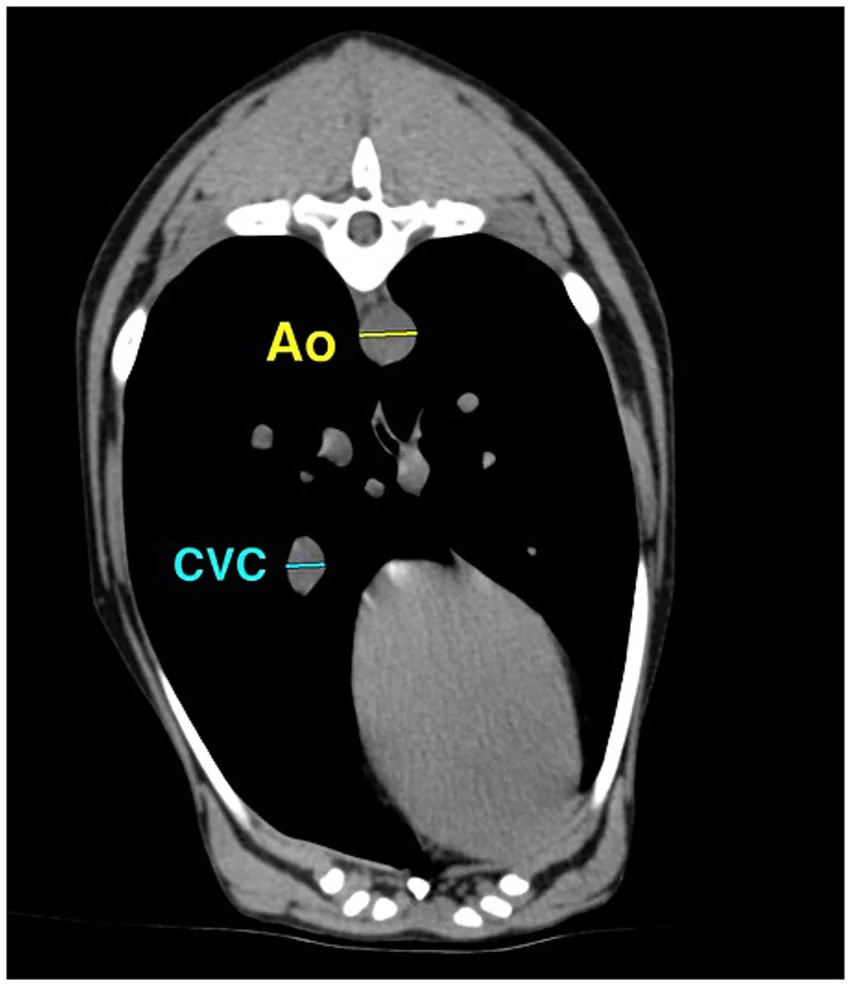
Transverse thoracic CVC and Ao measurements on computed tomography one of 34 whippets: these measurements were taken at the same slice for a given patient, between the heart and the diaphragm (not in contact with either), where the vessels were most sharply defined and their short axis was visually at their maximum length. Ao, aorta; CVC, caudal vena cava.

**Figure 2:**
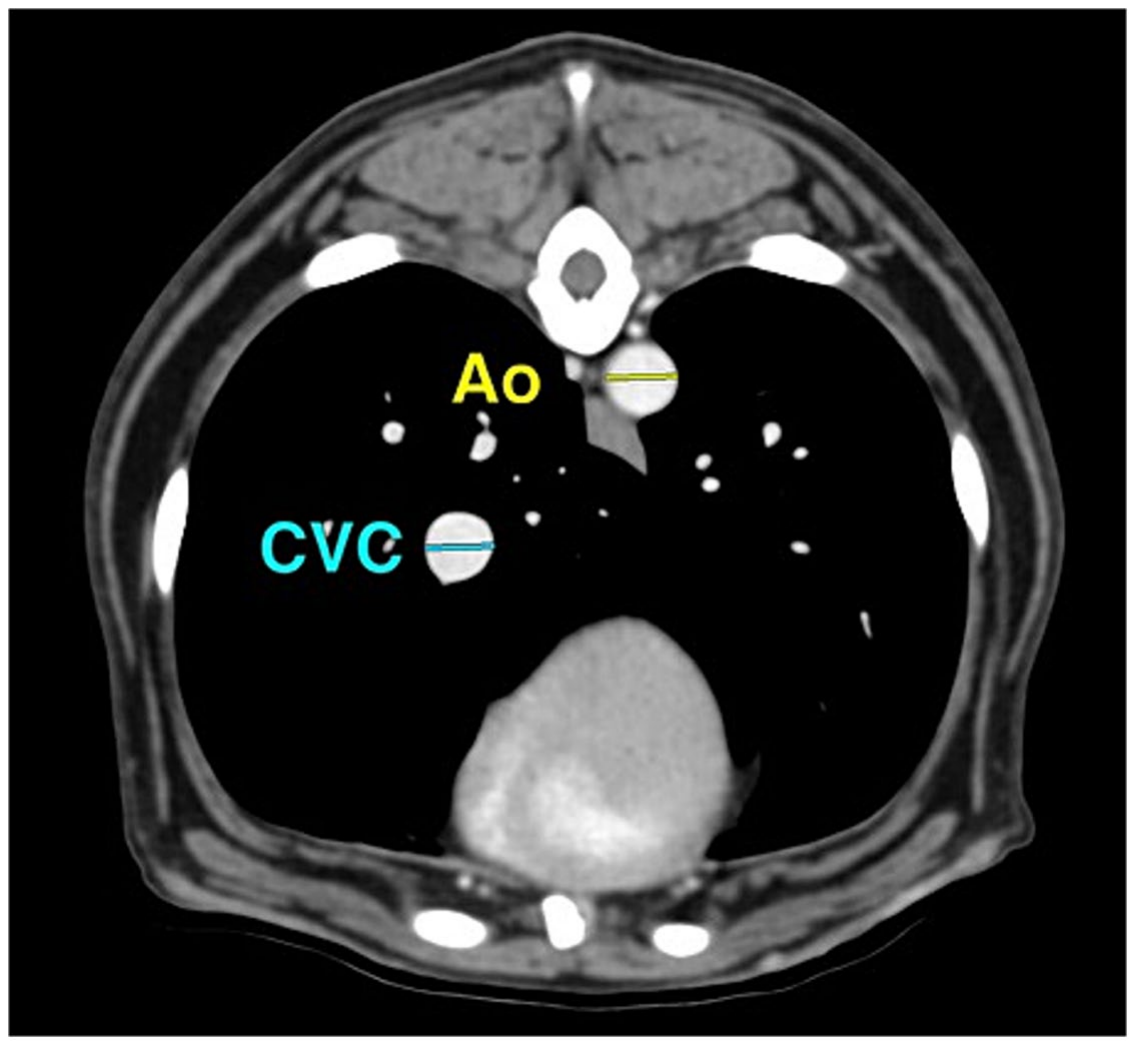
Transverse thoracic CVC and Ao measurements on computed tomography one of 34 whippets of various breeds: these measurements were taken at the same slice for a given patient, between the heart and the diaphragm (not in contact with either), where the vessels were most sharply defined and their short axis was visually at their maximum length. Ao, aorta; CVC, caudal vena cava.

**Figure 3:**
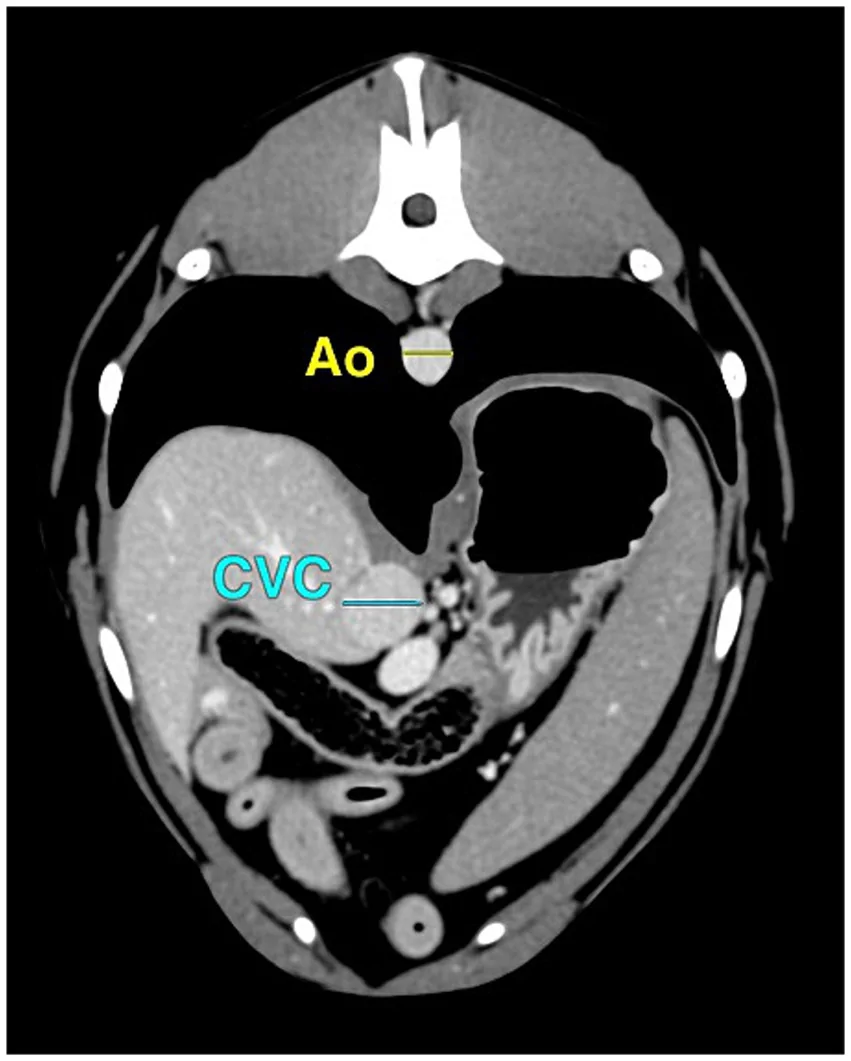
Transverse abdominal CVC and Ao measurements on computed tomography one of 34 whippets: these measurements were taken at the same slice for a given patient, between the post-renal segment and porta hepatis for the CVC and just cranial to the exit of the coeliac artery for the Ao, where the vessels were most sharply defined. Ao, aorta; CVC, caudal vena cava.

**Figure 4:**
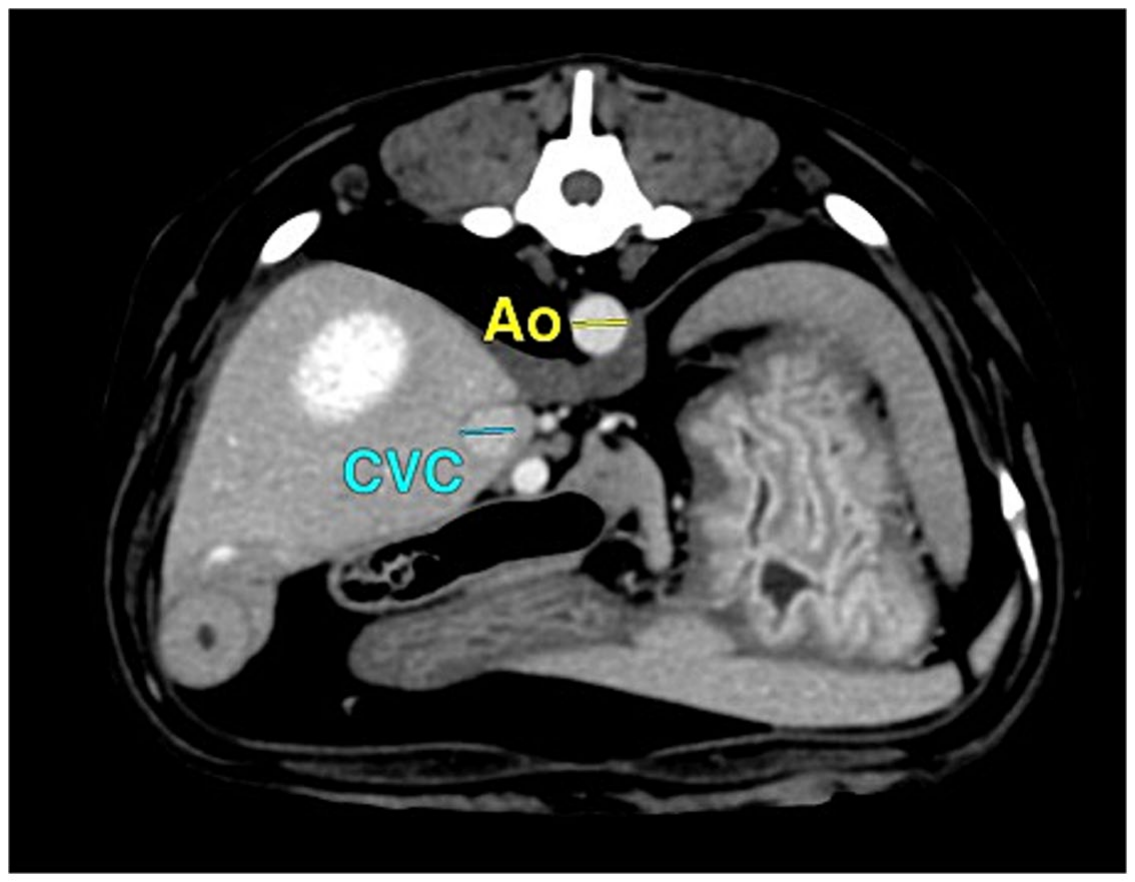
Transverse abdominal CVC and Ao measurements on computed tomography one of 34 whippets of various breeds: these measurements were taken at the same slice for a given patient, between the post-renal segment and porta hepatis for the CVC and just cranial to the exit of the coeliac artery for the Ao, where the vessels were most sharply defined. Ao, aorta; CVC, caudal vena cava.

### 2.4 Statistical analysis

CVC/Ao were calculated by dividing the CVC short axis diameters by the Ao diameters. Statistical analyses were performed using IBM SPSS Statistics (Version 29.0, IBM Corp., Armonk, NY, United States). Continuous data are presented as mean ± SD and median (IQR). Normality was confirmed for all four group–site distributions using the Shapiro–Wilk test (*W* = 0.94–0.98, all *p* > 0.05), supported by visual inspection of histograms and Q–Q plots. Whippets (*n* = 34) and control dogs (*n* = 34) were compared at each anatomical site (thorax, abdomen) using Welch’s two-sample *t*-test, and the abdominal and thoracic ratios were compared within each animal using paired t-tests; group differences are reported with 95% confidence intervals (CI) and Cohen’s d as a standardised effect size (*d*_z_ for paired comparisons). Group-specific mean ± 2 SD limits for the CVC/Ao were calculated. Because these were derived from a clinical population undergoing CT for a variety of non-cardiovascular conditions rather than from healthy dogs, and because the sample size (*n* = 34 per group) falls below the ASVCP-recommended minimum of 120 (10), they are reported as mean ± 2 SD limits observed in this cohort rather than formal reference intervals or breed-specific reference ranges.

Group means were also compared with the most recent reference for each imaging modality: for CT (6), each group was compared with the published value at the matching site using a Welch’s two-sample t-test from summary statistics (thorax 1.12 ± 0.23, abdomen 0.78 ± 0.25; *n* = 95); for ultrasonography (11), comparison was restricted to the abdomen and the proportion of dogs exceeding the upper limit of the published reference interval (0.93–1.32) was compared between groups using the Pearson’s chi-squared test without continuity correction. Because body weight and age both differed between groups, CVC/Ao was compared by analysis of covariance with body weight and age as covariates (homogeneity of slopes confirmed) and repeated within the weight range common to both groups (7.9–22.2 kg). Adjusted differences are reported with 95% confidence intervals and partial η^2^.

Comparisons were pre-planned and limited in number, so no correction for multiple comparisons was applied. As a sensitivity analysis, Holm’s step-down correction applied to the four comparisons with published reference values left all conclusions unchanged; only the whippet thoracic comparison would lose significance were all tests in the study treated as a single family, and that result is therefore interpreted as borderline. Intra and interobserver agreement were quantified using the intraclass correlation coefficient (ICC(A,1); two-way random effects, absolute agreement, single measures) with 95% CIs, and by Bland–Altman analysis of bias and 95% limits of agreement. Whether interobserver bias differed between groups was tested using Welch’s two-sample *t*-test on the per-dog differences. All tests were two-tailed with significance set at *p* < 0.05.

## 3 Results

### 3.1 Study populations

The study population included 34 whippets, selected from 82 that had undergone any CT examination; their mean age was 6 years 4 months at the time of the CT study (ranging from 4 months to 14 years 4 months). This group comprised fifteen females, three of which were entire, and nineteen males, six of which were entire. Their weights ranged from 7.9 kg to 22.2 kg, with a mean of 14.3 kg.

The 34 dogs in the control group were aged from 6 months to 15 years 3 months, with a mean age of 8 years 4 months. The different breeds were as followed: small cross breed (x5), Labrador (x3), Cocker Spaniel (x2), English Springer Spaniel (x2), French Bulldog (x2), Golden Retriever (x2), Labradoodle (x2), Border Collie, Border Terrier, Boxer, Dachshund, Dalmatian, English Setter, Fox Terrier, German Shepherd, Goldendoodle, Greyhound, Italian Spinone, Jack Russell Terrier, Lhasa Apso, Norwegian Buhund, Shih Tzu, and large cross breed. This control group comprised twelve females, two of which were entire, and twenty-two males, of which four were entire. Their weights ranged from 4.3 kg to 60 kg, with a mean of 22.2 kg.

Clinical indications and final diagnoses varied within each group. In whippets, the most common was rhinitis or sinonasal neoplasia (29.4%), with a smaller number having coagulopathy or immune-mediated thrombocytopenia; the remainder were imaged for tumour staging (thyroid, pharynx, neck or limb), pyrexia of unknown origin, neurological work-up, follow-up of previous pulmonary disease, or investigation of an inflammatory or infectious focus.

Most control dogs (67.6%) were scanned for tumour staging purposes with apocrine gland anal sac adenocarcinoma the most common primary neoplasm (21.7% of all tumours). Other indications included orthopaedic conditions, immune-mediated and inflammatory/infectious diseases.

### 3.2 Abdominal CVC/Ao

Whippets had significantly higher abdominal CVC/Ao than controls (1.54 ± 0.29, median 1.53 [IQR 1.30–1.73] vs. 1.28 ± 0.23; median 1.27 [IQR 1.14–1.39], mean difference 0.26, 95% CI 0.13–0.39; *p* < 0.001; *d* = 0.99) (Table 1). The full limits of abdominal CVC/Ao in whippets were from 1.04 to 2.10, and from 0.86 to 1.86 in control dogs.

**Table 1:** Mean CVC/Ao in whippets and control dogs at the abdominal and thoracic sites.

Site	Whippets, mean ± SD [median (IQR)]	Controls, mean ± SD [median (IQR)]	Mean diff (95% CI)	*p*	Cohen’s d
Abdomen	1.54 ± 0.29 [1.53 (1.30–1.73)]	1.28 ± 0.23 [1.27 (1.14–1.39)]	0.26 (0.13–0.39)	<0.001	0.99
Thorax	1.22 ± 0.23 [1.26 (1.13–1.35)]	1.28 ± 0.26 [1.26 (1.09–1.45)]	−0.06 (−0.18 to 0.06)	0.29	0.26

Whippets also had higher abdominal ratios than the previously published ultrasonographic reference interval for healthy conscious dogs (0.93–1.32) (11), with a significantly greater proportion of whippets than controls exceeding the upper limit of 1.32 (25/34, 73.5% vs. 14/34, 41.2%; risk difference 32.4 percentage points, 95% CI 8.8–51.3; Pearson chi-squared without continuity correction, *χ*^2^(1) = 7.28, *p* = 0.007; φ = 0.33, OR 3.97). They were likewise significantly larger than the most recent published CT values for anaesthetised dogs of various breeds and clinical conditions (abdominal CVC/Ao 0.78 ± 0.25) (6), with a mean difference of 0.76 (95% CI 0.65–0.87; *p* < 0.001; *d* = 2.9).

Control dogs also exceeded the ultrasonographic reference interval, with 14 of 34 (41.2%) above 1.32, and were also significantly higher than the published CT reference (mean difference 0.50, 95% CI 0.41–0.59; *p* < 0.001; *d* = 2.0), though to a lesser degree than whippets.

One of the randomly selected control dogs was a greyhound, a sighthound, but its abdominal ratio (1.33) lay close to the control group mean (1.28).

### 3.3 Thoracic CVC/Ao

Thoracic CVC/Ao did not differ significantly between whippets and controls (1.22 ± 0.23, median 1.26 [IQR 1.13–1.35] vs. 1.28 ± 0.26, median 1.26 [IQR 1.09–1.45]; mean difference −0.06, 95% CI −0.18 to 0.06; *p* = 0.29; d = 0.26) (Table 1). Compared with the most recent CT-derived values (thoracic CVC/Ao: 1.12 ± 0.23) (6), both groups had modestly but significantly higher thoracic ratios (whippets: mean difference 0.10, 95% CI 0.01–0.19, *p* = 0.034, *d* = 0.43; controls: mean difference 0.16, 95% CI 0.06–0.26, *p* = 0.003, d = 0.68). There are no published ultrasonographic values for the thoracic ratio as there is no acoustic approach to this segment of the CVC.

### 3.4 Effect of body weight and age

Whippets were lighter (14.3 ± 3.0 kg vs. 22.2 ± 13.7 kg; *p* = 0.002) and younger (6 years 4 months vs. 8 years 4 months; *p* = 0.031) than control dogs, so both variables were entered as covariates. After adjustment, whippets retained significantly higher abdominal CVC/Ao than controls (adjusted difference 0.29, 95% CI 0.15–0.42; *F* (1,64) = 17.55, *p* < 0.001, partial η^2^ = 0.215), while thoracic ratios remained comparable (adjusted difference −0.06, 95% CI −0.17 to 0.06; *p* = 0.362). Body weight was positively associated with CVC/Ao at both sites (abdomen *p* = 0.041; thorax *p* = 0.017), and age was negatively associated with the thoracic ratio (−0.023 per year; *F* (1,64) = 9.96, *p* = 0.002, partial η^2^ = 0.135) but not the abdominal ratio (*p* = 0.112). Restricting analysis to the weight range common to both groups gave near-identical results (abdomen: adjusted difference 0.32, 95% CI 0.15–0.49, *p* < 0.001; thorax: *p* = 0.918).

### 3.5 Site-specific differences and mean ± 2 SD

Within-animal comparison of the two anatomical sites reinforced the site-specific pattern. In whippets, the abdominal ratio was significantly higher than the thoracic ratio (mean difference 0.32, 95% CI 0.19–0.46; *p* < 0.001; *d*_z_ = 0.83), whereas in control dogs the abdominal and thoracic ratios were essentially identical (mean difference 0.00, 95% CI −0.09 to 0.09; *p* = 0.995; *d*_z_ = 0.00); the elevated ratio in whippets was therefore confined to the abdominal segment.

Preliminary mean ± 2 SD limits were derived for each group (Table 2). For the abdominal ratio, the whippet limit (0.96–2.13) lay above and only partially overlapped that of the control dogs (0.82–1.75), reflecting the higher abdominal ratios in whippets. As thoracic ratios did not differ between groups, either before or after adjustment, a single combined thoracic limit was derived from all 68 dogs (0.76–1.74). Given the sample size and the clinical nature of the study population, these values should be interpreted as the mean ± 2 SD observed in this cohort rather than as formal breed-specific reference intervals.

**Table 2:** Preliminary CVC/Ao ranges (mean ± 2 SD).

Site	Group	Preliminary CVC/Ao range
Abdomen	Whippets	0.96–2.13
Abdomen	Controls	0.82–1.75
Thorax	All dogs combined	0.76–1.74

As thoracic ratios did not differ between groups, a single combined range is reported for that site.

### 3.6 Inter and intra-observer agreement

Intraobserver agreement for the second observer was good to excellent (abdominal ICC 0.82–0.88; thoracic ICC 0.83–0.94). Interobserver agreement was excellent for the thoracic ratio (ICC 0.96, 95% CI 0.90–0.98) and moderate for the abdominal ratio (ICC 0.67, 95% CI 0.16–0.87). The first observer recorded higher abdominal ratios than the second by a mean of 0.13 (95% limits of agreement −0.18 to +0.45). This bias was of similar magnitude in whippets and controls (+0.17 vs. + 0.09; difference 0.08, *p* = 0.283), providing no evidence that it varied with breed group.

## 4 Discussion

The CVC/Ao is an easy-to-perform and well-established parameter in human and veterinary medicine on ultrasound (1, 11) and CT (6, 9), that provides insight into the fluid status of the patient (12–14). This study suggests that whippets have significantly larger abdominal CVC/Ao than non-whippet control dogs. A plausible explanation for this difference is breed-related physiological adaptations. Sighthound breeds, including whippets, have well-described cardiovascular adaptations (7, 8) associated with sprint athletic performance, including larger left ventricular dimensions and lower resting heart rates than non-sighthound dogs. Whippet-specific echocardiographic reference values have been proposed (15, 16) and it therefore stands to reason that whippet-specific CVC/Ao would be similarly useful.

Interestingly, thoracic CVC/Ao were comparable between the two groups. A direct within-animal comparison of the two anatomical sites confirmed that this regional difference was real and not an artefact of between group variations: in whippets the abdominal ratio was significantly higher than the thoracic ratio, whereas in control dogs the two segments were equivalent. The elevation in whippets was therefore confined to the abdominal segment rather than reflecting a generalized increase in CVC calibre. We speculate that this regional difference may relate to anatomical constraints within the thorax (diaphragm, mediastinum and right atrium), where the CVC may be less able to dilate than in the abdomen, in which increased venous return could be accommodated more readily; this suggestion is extrapolated from human data (17, 18) and remains to be confirmed in dogs.

Two observations temper the interpretation of this finding. Firstly, an elevated ratio may arise from a larger CVC, a smaller Ao, or both, and the present study reports only the ratio; whether the difference reflects greater venous capacitance or breed-related aortic dimensions therefore remains undetermined, and studies reporting absolute vessel dimensions indexed to body size are needed to resolve this. Secondly, one of the randomly selected control dogs was a greyhound, also a sighthound, yet its abdominal ratio (1.33) lay close to the control group mean (1.28). Although a single animal permits no inference, this raises the possibility that the finding is specific to whippets rather than common to sighthounds, and studies including other sighthound breeds would help clarify whether the difference is breed or group-related.

In addition to the between-group comparison, we derived preliminary mean ± 2 SD limits for the CVC/Ao (Table 2). For the abdominal ratio, the whippet limit (0.96–2.13) lay largely above that of the control dogs (0.82–1.75), whereas thoracic ratios did not differ between groups and a single combined limit (0.76–1.74) is therefore reported. These limits should be interpreted with caution: the sample size (*n* = 34 per group) falls below the number recommended for formal reference-interval estimation (10), and the limits were derived parametrically rather than by the preferred non-parametric method. Nonetheless, they provide initial breed-associated set of limits against which future whippet CT studies may be compared and reinforce the principle that reference values generated from heterogeneous, multi-breed populations may not be transferable to some breeds.

The two groups had abdominal CVC/Ao that exceeded the most recent published values for both imaging modalities. Compared with the most recent CT reference values, derived from anaesthetised dogs of various breeds and clinical conditions (abdominal CVC/Ao 0.78 ± 0.25) (6), both groups again had significantly higher ratios (whippets, mean difference 0.76, *p* < 0.001; controls, mean difference 0.50, *p* < 0.001), the difference being greater in whippets. Both whippets and control dogs also had thoracic CVC/Ao significantly higher than the most recent CT values from anaesthetised dogs (6).

Hanazono et al. demonstrated that several anaesthesia-related and patient-related factors influence the dimensions of the CVC on CT, including mean arterial pressure, intra-abdominal pathology, anaemia, and cardiac disease (6). These factors, together with potential differences in body weights, ages and breeds between our control group and the previous cohorts may at least partly explain the discrepancy. The underlying disease and diagnosis of any given patient may also influence vascular dimensions, particularly the CVC diameter, and alter the ratios accordingly (19–21).

Compared with the ultrasonographic reference interval established in healthy conscious dogs (0.93–1.32) (11), 25 of 34 whippets (73.5%) and 14 of 34 control dogs (41.2%) lay above the upper limit, while only 2 controls (5.9%) and no whippets fell below the lower limit; the proportion exceeding the upper limit was significantly greater in whippets than in controls (*p* = 0.007).

The direct application of ultrasonographic CVC/Ao reference values from conscious dogs to anaesthetised patients imaged with CT under general anaesthesia or of CT-derived ratios that disregard breed is therefore not recommended. To our knowledge, this is the first study to report breed-associated differences in CT-derived CVC/Ao ratios.

Some of the measurements in whippets were obtained pre-contrast whereas all control measurements were post-contrast (Figures 1–4). Extrapolating from humans, the hyperosmolar contrast medium administration itself can transiently expand intravascular volume and cause vasodilation, therefore a small effect on measured CVC and/or Ao dimensions cannot be excluded (22). The fluid boluses commonly used to mitigate acute kidney injury and flush the contrast medium could also affect vascular dimensions. The CVC, as a thin-walled high-compliance capacitance vessel, would be expected to distend more readily than the Ao in response to any contrast-induced increase in intravascular volume, which could preferentially increase the measured CVC/Ao. The whippet measurements post-contrast could potentially therefore be even larger than pre-contrast which would widen the already existing difference between both groups. The exact effect of non-ionic iodinated contrast medium on vascular dimensions has not yet been studied on dogs however, this represents a plausible explanation that requires further validation.

The higher abdominal ratio in whippets was not explained by differences in body size or age. Whippets were both lighter and younger than the control dogs, but because CVC/Ao increased with body weight, their smaller size would have been expected to lower rather than raise their ratios; adjustment for both covariates therefore increased rather than diminished the between-group difference, as did restriction to the weight range common to both groups. The elevated abdominal ratio in whippets is therefore most plausibly a breed-related characteristic rather than an artefact of differing population composition. Body weight nonetheless remained associated with the CVC/Ao at both sites, indicating that the ratio does not entirely normalise for body size, and the thoracic ratio declined with age independently of breed and body weight. The ratio assumes an approximately linear relationship with body size, whereas the linear dimensions of blood vessels scale non-linearly (allometrically) with body mass; an analysis of covariance using raw body weight as a covariate may therefore under-adjust at the extremes of the weight range. Ratiometric scaling is clinically practical but does not fully remove size dependence, and future large-scale, multi-breed studies employing allometric modelling would help to establish truly size-independent vascular reference standards. Both observations suggest that reference intervals for the CVC/Ao may need to account for body size and age as well as breed and imaging modality.

From a clinical perspective, an abdominal CVC/Ao above the published ultrasonographic upper limit should therefore not, on its own, be interpreted as evidence of volume overload or right-sided heart disease in an anaesthetised whippet (20).

There are some limitations to this study, with the first being the small sample size in each group; whippets are not a common breed in our patient population. The retrospective nature of the study and the lack of standardised anaesthesia protocols are also important limitations, meaning the specific effects of anaesthesia on patients haemodynamics, and consequently on the CVC and Ao sizes, could not be properly assessed (6, 18). Another limitation is the use of two different CT scanners over the period of the study, unevenly distributed between the two groups, offering different spatial resolutions. Although acquisition settings were not formally standardised, the pre-set protocols of both scanners were similar (including kVp, mAs, timing of contrast), partially reducing the impact of this limitation. Additionally, the site-specificity of our finding makes a scanner-related contribution unlikely (confined to the abdomen), as an instrumental bias would be expected to affect both anatomical sites; such a contribution cannot be entirely excluded however. The breath-hold technique also differed between the groups (see Materials and Methods); however, the inspiratory positive-pressure hold used predominantly in the control group would be expected to raise intrathoracic pressure at least as much as the end-expiratory hold used in most whippets, so any resulting CVC congestion would tend to raise control ratios and thereby narrow, rather than widen, the observed whippet–control difference. A further limitation is that dogs were not excluded based on intra-abdominal disease, which can alter CVC diameter by reducing the venous return. On review of the diagnoses, space-occupying or potentially pressure-relevant abdominal and retroperitoneal lesions were, if anything, more frequent in the control group than in the whippets, so this factor too would be expected to narrow rather than widen the observed difference; nonetheless, a contribution from unrecognised or subclinical abdominal disease cannot be excluded, and prospective studies restricted to dogs without abdominal disease would be needed to exclude it entirely. Another shortcoming of this study is the fact that all measurements were initially performed by a single observer not blinded to group assignment. To address this, a random subset of 20 dogs was re-measured twice by a second, blinded observer. Agreement was excellent for the thoracic ratio and moderate for the abdominal ratio, the latter showing a systematic difference between observers of approximately 0.13. Importantly, this difference was of similar magnitude in both groups, indicating a difference in measurement technique rather than bias introduced by knowledge of breed; because it applies equally to whippets and controls, it would not generate the observed between-group difference. Nonetheless, the reliability subset comprised only 10 dogs per group, and a modest degree of differential bias cannot be excluded. The moderate abdominal agreement probably reflects the length of the abdominal segment over which the CVC was measured and supports the use of a more tightly defined landmark in future studies. Lastly, as discussed above, the CVC/Ao does not fully correct for body size (within controls, *r* = 0.38, *p* = 0.028), although adjustment for body weight increased rather than diminished the observed difference.

Prospective studies in targeted breed groups, using standardized CT acquisition (including presence or absence of iodinated contrast medium administration) and anaesthetic protocols (including blood pressure and echocardiographic data) are needed to establish more robust breed-specific CVC/Ao ratios in dogs without systemic diseases.

## 5 Conclusion

Whippets appear to have significantly larger abdominal CVC/Ao on CT than non-whippet controls, whereas thoracic ratios are comparable between the two groups; the within animal comparison confirmed that this elevation is confined to the abdominal segment rather than reflecting a generalised increase in CVC size. Both groups had abdominal ratios that exceeded the published ultrasonographic reference interval as well as the most recent CT-derived values from anaesthetised dogs of mixed breeds, indicating that neither ultrasonographic nor multi-breed CT reference intervals can be reliably applied to CT studies in whippets. The mechanism underlying the regional difference remains to be established, and studies reporting absolute CVC and Ao dimensions in other breeds are needed. On this basis, we provide preliminary mean ± 2 SD limits for the whippet abdominal ratio (0.96–2.13) and combined thoracic limit (0.76–1.74), which should be regarded as cohort observations rather than formal reference ranges, given the limited sample size and the clinical nature of the study population (Table 2).

## Data Availability

The original contributions presented in the study are included in the article, further inquiries can be directed to the corresponding author.

## Footnotes

1 Iohexol: Omnipaque, 300 mg I/mL, 2 mL/kg, GE Healthcare, Chicago, IL, USA.

2 Dexmedetomidine: Dexdomitor, 0.5 mg/mL, Vetoquinol, UK.

3 Methadone: Comfortan, 10 mg/mL, Eurovet Animal Health BV, The Netherlands.

4 Butorphanol: Butorphanol tartrate, 14.58 mg/mL, Zoetis, UK.

5 Alfaxalone: Alfaxan, 10 mg/mL, Zoetis, UK.

6 Propofol: Propoflo, 200 mg/20 mL (10 mg/mL), Zoetis, UK.

7 Ketamine: Ketamidor, 100 mg/mL, Chanelle Vet UK Ltd., UK.

8 Diazepam: Ziapam, 5 mg/mL, TVM UK Animal Health Ltd., UK.

9 Isoflurane: Hospira Inc., Lake Forest, IL 60045, United States.
